# Electroacupuncture alleviates retrieval of pain memory and its effect on phosphorylation of cAMP response element-binding protein in anterior cingulate cortex in rats

**DOI:** 10.1186/s12993-015-0055-y

**Published:** 2015-03-04

**Authors:** Jing Sun, Xiao-mei Shao, Fang Fang, Zui Shen, Yuan-yuan Wu, Jian-qiao Fang

**Affiliations:** Department of Neurobiology and Acupuncture Research, The Third Clinical Medical College, Zhejiang Chinese Medical University, Hangzhou, China

**Keywords:** Electroacupuncture, Pain memory, Phosphorylation of cAMP response elment-binding protein, Anterior cingulate cortex, Rat

## Abstract

**Background:**

Recent evidence suggests that persistent pain and recurrent pain are due to the pain memory which is related to the phosphorylation of cAMP response element-binding protein (p-CREB) in anterior cingulate cortex (ACC). Eletroacupuncture (EA), as a complementary Chinese medical procedure, has a significant impact on the treatment of pain and is now considered as a mind-body therapy.

**Methods:**

The rat model of pain memory was induced by two injections of carrageenan into the paws, which was administered separately by a 14-day interval, and treated with EA therapy. The paw withdrawal thresholds (PWTs) of animals were measured and p-CREB expressions in ACC were detected by using immunofluorescence (IF) and electrophoretic mobility shift assay (EMSA). Statistical comparisons among different groups were made by one-way, repeated-measures analysis of variance (ANOVA).

**Results:**

The second injection of carrageenan caused the decrease of PWTs in the non-injected hind paw. EA stimulation applied prior to the second injection, increased the values of PWTs. In ACC, the numbers of p-CREB positive cells were significantly increased in pain memory model rats, which were significantly reduced by EA. EMSA results showed EA also down-regulated the combining capacity of p-CREB with its DNA. Furthermore, the co-expression of p-CREB with GFAP, OX-42, or NeuN in ACC was strengthened in the pain memory model rats. EA inhibited the co-expression of p-CREB with GFAP or OX-42, but not NeuN in ACC.

**Conclusions:**

The present results suggest the retrieval of pain memory could be alleviated by the pre-treatment of EA, which is at least partially attributed to the down-regulated expression and combining capacity of p-CREB and the decreased expression of p-CREB in astrocytes and microglia cells.

## Background

Persistent pain caused by inflammation, surgical wounds, and other injuries is a very common phenomenon. Patients who suffer from nociceptive pain for a long time may feel persistent pain even after recovery of the initial cause of pain [1,2]. Animal studies also reported that nociceptive hyperalgesia could recur far away from the primary injured region after repeated carrageen-induced inflammation [3]. Recent researches indicated the pain memory was a key mechanism of this special pain. The initial memory of the pain can be accentuated by the emotional component of reliving its memory, resulting in a more vivid and stronger memory of the pain [4]. The pain syndrome, due to synaptic plasticity changes in the central nervous system, existed after the recovery of primary inflammation [5]. So it has been suggested that if pain memory itself is a primary etiology of persistent pain, how to inhibit the memory of pain become an effective way of analgesia for prolonged pain.

Which part of the brain participates in this “pain memory”? Evidence suggests that the anterior cingulate cortex (ACC), the insular cortex, and the amygdala are examples of regions implicated in both pain and memory [4]. The ACC is an important site for cortical regulation of nociception and persistent pain after amputation [6]. Furthermore, based on functional magnetic resonance imaging (fMRI) studies in both rats and humans, the ACC is involved in pain memory processing [7,8].

The transcription factor cAMP response element-binding protein (CREB) plays an important role in learning and memory. Evidence from aplysia, drosophila, mice, and rats all showed that CREB-dependent transcription was required for the cellular events underlying long-term memory, especially the late phase of long-term potentiation (LTP) [9-11]. A variety of neuron types,especially the neurons and astrocytes, are able to activate CREB to participate in the formation of synaptic plasticity in the central nervous system and the regulation of physiological and pathological processes [12,13]. The phosphorylation of CREB is directly related to the process of acquisition, consolidation, and retrieval of pain memory [14,15].

Electroacupuncture (EA), a widely used Chinese medical therapy, is a modified form of acupuncture that uses electrical stimulation. Several studies have investigated the effectiveness of EA on pain induced by inflammation [16-19]. EA has also been proven to enhance the function of cognition, learning, and memory in animal models of ischemic injury, vascular dementia, and Alzheimer’s disease [20-23]. Some researchers support the notion that acupuncture is a mind-body therapy that helps to reintegrate important neural dimensions of inner life and to establish psychophysical pain homeostasis [24]. However, few studies have investigated whether EA stimulation is effective for pain memory and elucidated the underlying mechanisms of this treatment.

Therefore, in the present study, we aimed to determine whether EA could ameliorate pain memory by regulating the expression and combining capacity of phosphorylation of CREB (p-CREB) as well as the co-expression of p-CREB in neurons and gliocytes in the ACC.

## Methods

### Animals

Thirty male adult Sprague–Dawley rats (Sino-British SIPPR/BK Lab. Animal Ltd., Shanghai, China) weighting 180–200 g (6 weeks) were group housed for every five rats per cage in a controlled room temperature (22°C) and kept on a 12 h light–dark cycle with free access to rodent chow and water. The total number of rats was calculated by the estimation of sample size based on comparing multiple samples mean with the power of 95%. At least 6 rats were needed in each group revealed by the calculated results. To reduce the results error, the final sample size was determined 10 rats of each group. Thirty rats were randomly divided into 3 groups (n = 10 each): control group, model group and EA group. From each group, ten rats were all used for the detection of paw withdrawal thresholds (PWTs). Three of 10 rats were further used for the determination of p-CREB combinative activity in ACC by electrophoretic mobility shift assay (EMSA), while seven others were used for the measurement of expression of p-CREB in nerve cells of ACC by immunofluorescence (IF). All animal experiments were performed according to the National Institutions of Health Guide for the Care and Use of Laboratory Animals [17].

### Establishment of pain memory model

We used the model of pain memory described by Igor Kissin [3], in which inflammation was induced by two injections of carrageenan. The first inflammation was induced by subcutaneous injection (0.5 mm diameter × 20 mm long needle) of 0.1 mL of 2% carrageenan (Sigma Chemical Co, St. Louis, MO, USA) into the plantar surface of the right hind paw. The plantar surface of the rat’s left hind paw was injected 14 days after the first injection to the right hind paw, giving rise to the second inflammation.

The response to noxious pressure was determined by measuring paw mechanical withdrawal thresholds. Nociceptive memory regarding secondary hyperalgesia was assessed by measuring distant hyperalgesia, in which the previously injected hind paw (right) didn’t receive another injection when repeated carrageenan-induced inflammation was made in the left hind paw. Therefore, the right hind paw and left ACC of rats were used for the detection of pain memory in this study.

### EA treatment procedures

The rats of the EA group were administrated electroacupuncture stimulation as previously described [17,25]. The treatment was given at 5 h (after the behavioral test), 23 h (before the behavioral test), 47 h (before the behavioral test), and 71 h (before the behavioral test) after the first carrageenan-injection for 30 min per treatment. The stainless acupuncture needles (0.3 mm in diameter × 13 mm in length) and HANS Acupuncture Point Nerve Stimulator (LH-202H; Huawei Co., Ltd., Beijing, China) were applied at bilateral acupoints “Zusanli” (ST36, 7 mm lateral to the anterior tubercle of the tibia) and the reference electrode was fixed at 1 cm inferior “Zusanli” in rats. The parameters of EA were set as follows: 2/100 Hz of frequency with automatically shifting between 2 Hz and 100 Hz stimulation for 3 s each; a square wave current output (pulse width: 0.2 ms); an intensity range of about 1–2 mA adjusted to animals’ local muscle contractions. The EA administration and other managements were conducted by an acupuncture specialist. Animals were calmed by placing the heads in black hoods with no physical restraint. No sign of stress, such as increased urination or defecation, was observed. At the same time, the rats of both model and control groups also received the same administration to maintain consistency.

### Behavioral test procedures

The PWTs were the nociceptive test used to provide behavioral evidence in this study. The response to noxious pressure was detected by UGO-Basile Dynamic Plantar Aesthesiometer (UGO 37450, Milan, Italy). Ten rats of each group were included to assess the pain situation and were measured at 4 h, 24 h, 48 h, and 72 h after the different two injections. As described before [26,27], rats were placed on a metal mesh table and in an individual plexiglass housing designed to provide adequate comfort and ventilation for 30 min to adapt to the environment prior to starting experimental manipulations. A steel rod (0.5 mm diameter) was pushed up to the plantar surface of the hind paw with increasing force (2.5 g/s). The cutoff pressure was 50 g. The thresholds were recorded when the rat struggled with increasing pressure or reached the cutoff pressure. Five measurements were taken at 5-min intervals, and the last four were averaged. To reduce the stressful stimulation of environments, all behavioral tests were done between 9:00 and 15:00 by the same investigator, and the rats were habituated to the testing procedures and environments 2 days before the experiment. The investigator was blind to the rats’ allocation in grouping.

### Electrophoretic mobility shift assay procedures

Nuclear proteins for EMSA were extracted using the Nuclear and Cytoplasmic Extraction Kit (Beyotime, Shanghai, China). Sequences corresponding to the CRE-binding site labeled with biotin (synthesized and 3’ labeled by Sangon Inc., Shanghai, China) of oligonucleotide probes were as follows: 5’-AGA GAT TGC CTG ACG TCA GAG AGC TAG-3’ and 5’-CTA GCT CTC TGA CGT CAG GCA ATC TCT-3’. To determine the specificity of the binding complexes of the proteins of p-CREB and its DNA, reactions that using unlabeled probes and unlabeled mutational probes were performed competitively. The sequences of unlabeled p-CREB probes were as the same as biotin-labeled p-CREB probes while unlabeled mutational probes’ sequences were 5’-AGA GAT TGC CTG TGG TCA GAG AGC TAG-3’ and 5’-CTA GCT CTC TGA CCA CAG GCA ATC TCT-3’.

EMSA was performed by using the Light Shift Kit (Pierce, Thermo, Rockford, IL, USA). According to the protocols of the kit, the binding reaction was carried out in a total volume of 20 mL containing ultrapure water, 10× binding buffer, 1 μg/μL Poly (dl•dc), 50% glycerol, 1% NP-40, 100 mM MgCl2, unlabeled p-CREB probes/unlabeled mutational p-CREB probes, protein extract (15 μg/lane), and biotin-labeled p-CREB probes. Samples were incubated on ice for 50 min, and mixed with 5× loading buffer subsequently. The complexes of protein and oligonucleotide probes were loaded in a native 6% polyacrylamide gel and run in 0.5× TBE buffer, 100 V for 1 h, then transferred onto a positively charged nylon membrane at 380 mA for 30 min. After the membrane was crosslinked, we proceeded directly to detection by Chemiluminescent Nucleic Acid Detection Module (Pierce, Thermo, Rockford, IL, USA). For competitive experiments, the unlabeled competitive probes which were 50-fold excess to the labeled probes were pre-incubated for 20 min.

### Double-immunofluorescence labeling procedures

Left ACC of rats’ brain in each group used for IF was fixed and post-fixed in 4% paraformaldehyde (pH 7.3), dehydrated in 15–30% gradient sucrose and frozen at −80°C. Coronal brain slices containing the ACC were cut at 30 μm on a cryostat microtome (Microm HM 550; Thermo). Sections at the level of bregma 3.2 mm were chosen from the bregma 3.2 mm, 2.7 mm, and 2.2 mm by preliminary experiments. According to the double-immunofluorescence labeling procedures [18,21], coronal brain slices were washed with 0.01 M PB saline (PBS, pH 7.4) for 2 × 5 min and blocked with 5% goat serum in PBS for 1 h at 37°C. The sections were then incubated overnight at 4°C with an anti-phospho-CREB (Ser133) polyclonal antibody (rabbit-anti, 1:1000; Millipore, Billerica, MA, USA) and either an anti-GFAP monoclonal antibody (mouse-anti, 1:100; Abcam, Boston, MA, USA), an anti-FOX3/NeuN monoclonal antibody (mouse-anti, 1:1000; Abcam), or a CD11b monoclonal antibody (mouse-anti, 1:100; AbDSerotec, Oxford, UK). After 3 × 10 min rinses in PBS, the sections were incubated in a Cy3-conjugated secondary antibody (goat anti-rabbit, 1:1000, Jackson ImmunoLabs, West Grove, PA, USA) and an Alexa Fluor 488-conjugated secondary antibody (goat anti-mouse, 1:200; Jackson ImmunoLabs). Finally, the slides were examined with a Nikon A1R laser scanning confocal microscope and the positive cells were counted with NIS elements AR software, which is equipped for Nikon DS series digital cameras in laser confocal microscope to control microscope and capture microscopic image. This software is also used for immunofluorescence data analysis.

### Statistical analysis

Statistical comparisons among different groups were made by one-way, repeated-measures analysis of variance (ANOVA). For the following multiple comparisons, the least significant difference (LSD) and the Dunnett’s test were used for equal variances assumed or not assumed respectively, as examined by the homogeneity of variance test. Data were expressed as means ± SEM. Significance was determined at the level of P < 0.05.

## Results

### EA intervened in the mechanical allodynia in the pain memory model

**Figure 1:**
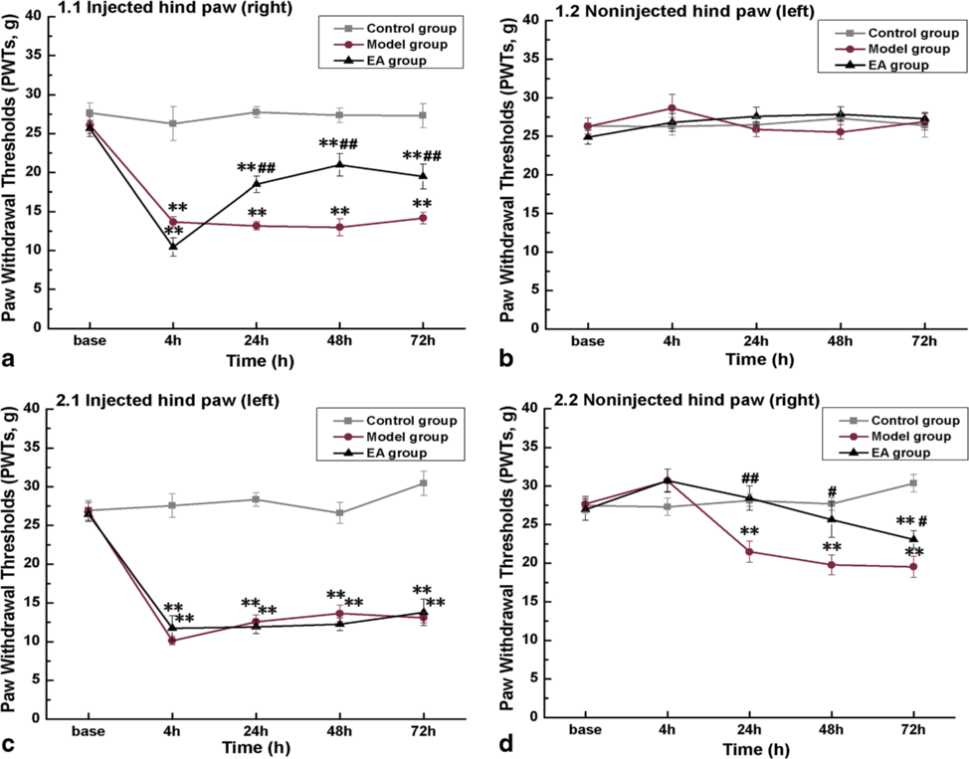
**Mechanical allodynia of rats in repetitive-crossover carrageenan injection with a 2-week interval.** Rats of each group were tested for mechanical allodynia of bilateral hind paws at time points of before (Base), and 4 h, 24 h, 48 h, 72 h after carrageenan-injection. The first injection was administered into the plantar surface of the right paw **(a and b)**; the second injection was administered into the left paw 2 weeks later **(c and d)**. The results showed a sustained decrease of mechanical threshold in the initial injected paw **(d)**. Error bars indicated standard error of the mean. Ten rats for each group. ** p < 0.01, vs. the control group; ^#^ p < 0.05, ^##^ p < 0.01, vs. the model group.

We used the rat model of pain memory described by Igor Kissin [3]. Following the model establishment and testing of mechanical allodynia (Figure 1), we investigated the changes in PWTs after two carrageenan injections separated by a 14-day interval. At 4 h after the first injection of carrageenan, the injected hind paw showed a profound decrease in noxious withdrawal thresholds compared with the control group (P < 0.01), keeping decrease until 72 h. While EA intervention, administrated at 5 h after carrageenan injection and for continuous 4 treatments with 24 h interval, increased the pain threshold at hours of 24, 48 and 72, compared with the model group (P < 0.01) (Figure 1a). No significant changes in PWTs at the observed times were found on the non-injected hind paw (Figure 1b).

Fourteen days after the pain withdrawal thresholds had returned to its baseline, the second injection of carrageenan to the previously non-injected hind paw caused a similar decrease of PWTs in the left hind paw from 4 h to 72 h. The pre-administration of EA did not produce any change in PWTs compared with the model group at any time point (Figure 1c). While in the right hind paw, the one previously injected and non-injected twice, PWTs in the model rats did not obviously change at 4 h, but were decreased from the baseline of 27.68 ± 0.97 g to 21.46 ± 1.36 g (P < 0.01) at 24 h, 19.77 ± 1.26 g (P < 0.01) at 48 h and 19.51 ± 1.36 g (P < 0.01) at 72 h (Figure 1d). At the same time, compared with the model group, EA intervention kept higher PWTs at 24 h (P < 0.01) and 48 h (P < 0.05), sharing similar level in PWTs with the control group. The PWTs at 72 h were decreased in EA group compared with the control group (P < 0.05), but it still showed higher than that in the model group (P < 0.05). The PWTs at 24 h, 48 h and 72 h in control group showed no difference compared with its baseline.

### EA reduced the phosphorylation level of CREB in ACC

**Figure 2:**
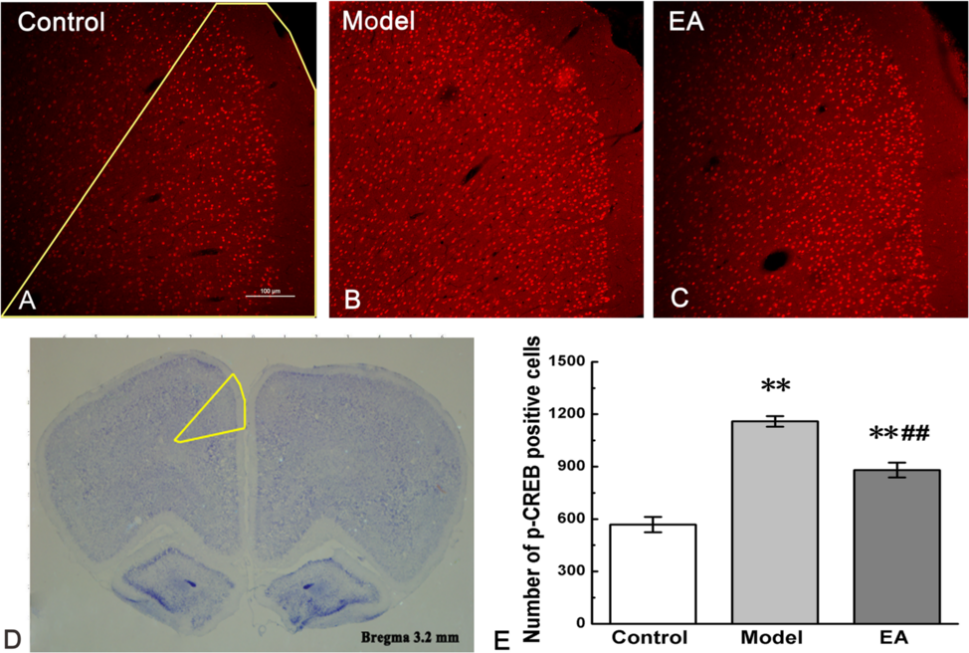
**Phosphorylation of CREB in the anterior cingulate cortex (ACC).** Differences in p-CREB expressions in control, model, and EA groups were presented in figure **a**, **b**, and **c**. Yellow line in figure a indicated the selected field in which the numbers of p-CREB-positive cells were counted. The left ACC in the harvested brain cortex was circled with a yellow line in figure **d**. Quantification of p-CREB positive cells was shown in figure **e**. Error bars indicated standard error of the mean. Three rats for each group, five slides for each rat. ** p < 0.01, vs. the control group; ^##^ p < 0.01, vs. the model group.

**Figure 3:**
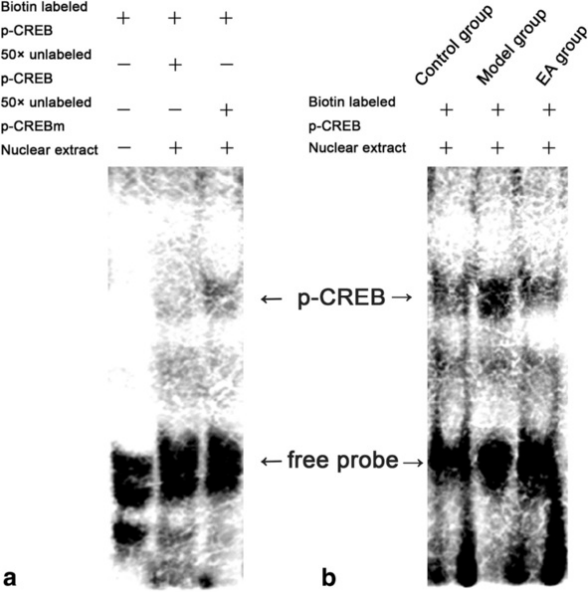
**Binding activity of p-CREB in nuclear extracts assessed by EMSA.** The part **a** showed the excess of unlabeled p-CREB probes and unlabeled mutational p-CREB probes abolished binding, demonstrating the specificity of all binding complexes. Binding activity of p-CREB among the control, model, and EA groups was shown in the part **b**. Arrows indicated the binding of p-CREB and the free probe.

CREB is one of the most important nuclear transcription factors involved in regulating memory function. We tested the activation of p-CREB in the ACC of the pain memory model with IF and EMSA. Figures 2 and 3 showed the results separately. The left ACC, which reflected the change of original injected paw (right) [28], was chosen for testing. Numbers of p-CREB positive cells were increased in the rats of model group compared with those in the control group (P < 0.01). In the EA group, the positive cells of p-CREB were more than those in the control group (P < 0.01), but less than those in the model group (P < 0.01).

EMSA performed with a CRE-containing oligonucleotide revealed different binding complexes attributed to electrophoretic mobility. The binding complexes observed in the polyacrylamide gel were of specific sequence because the retarded bandings were lost when the 50× unlabeled p-CREB probes and 50× unlabeled mutational p-CREB probes were used (Figure 3a). EMSA was performed by using the p-CREB proteins with biotin-labeled probes that containing p-CREB DNA among different groups. The observed binding complexes showed different combining capacities of p-CREB among the control, model, and EA groups. The band of model group was much stronger than that of control group, while EA attenuated the band as compared with model group (Figure 3b).

### Activation of astrocytes, microglia, and neurons in pain memory model and the declining of co-localizations of p-CREB with GFAP, OX-42, but not NeuN in ACC by EA

To further clarify the localization of p-CREB in ACC after formation of pain memory and cellular targets of EA treatment, we examined the activations of astrocytes (GFAP), microglia (OX-42) and neurons (NeuN) as well as the co-localizations of p-CREB in these cell types in ACC.

**Figure 4:**
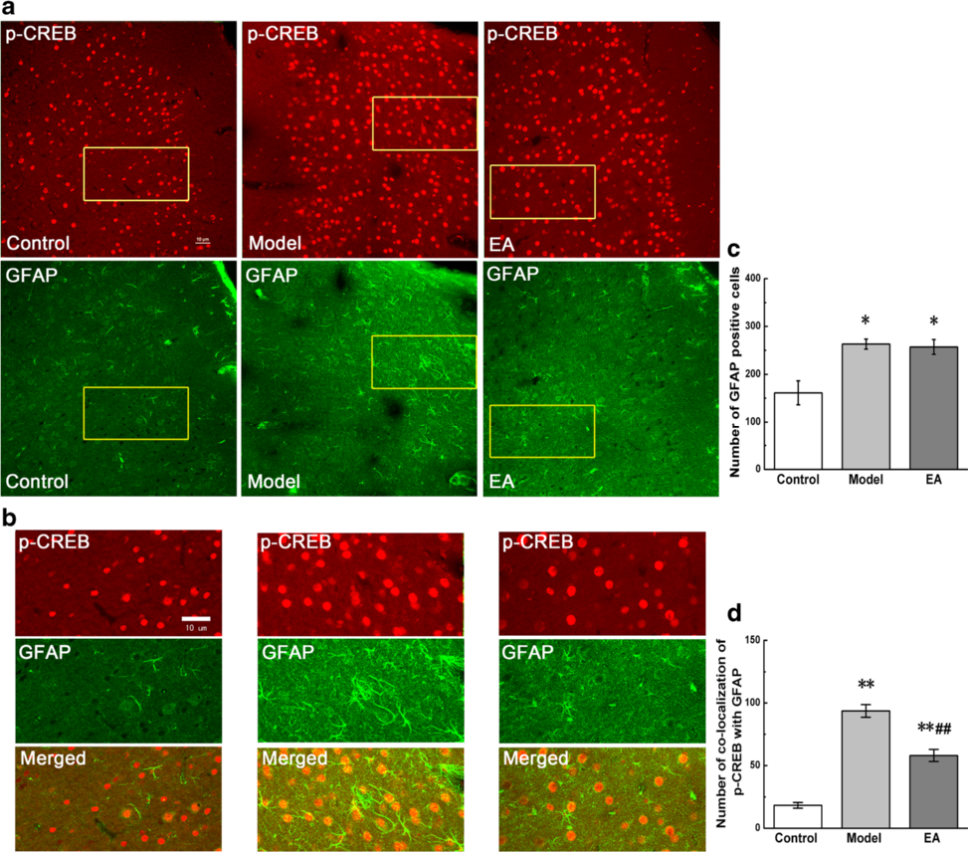
**Co-localization of p-CREB and GFAP in coronal brain sections of ACC.** Photomicrographs showed the expression of p-CREB (red) and GFAP (an astrocytic marker, green) from the same sections in figure **a**. Figure **b** was a high magnification image of the areas indicated by the yellow squares in the figure **a**. The double-immunofluorescence labeling showed that p-CREB co-expressed with GFAP in the ACC. Numbers of GFAP-positive cells and co-localization of p-CREB with GFAP were analysed in figure **c** and **d**. Error bars indicated standard error of the mean. Four rats for each group, five slides for each rat. * p < 0.05, ** p < 0.01 vs. the control group; ^##^ p < 0.01 vs. the model group.

The results from IF showed a substantial increase in the number of GFAP-positive cells after retrieval of pain memory, compared with that in the control group (P < 0.05). EA treatment produced no obvious decrease of GFAP-positive cells in ACC compared with the model group (Figure 4a, c). The double immunofluorescence examinations showed remarkably increased numbers of GFAP-positive cells co-expressing p-CREB in the model group, compared with those in the control group (P < 0.01). In contrast to the lack of changes in GFAP immunostaining after EA intervention, the decreased numbers of GFAP-positive cells co-expressing p-CREB were observed, compared to those in the model group (P < 0.01) (Figure 4b, d).

**Figure 5:**
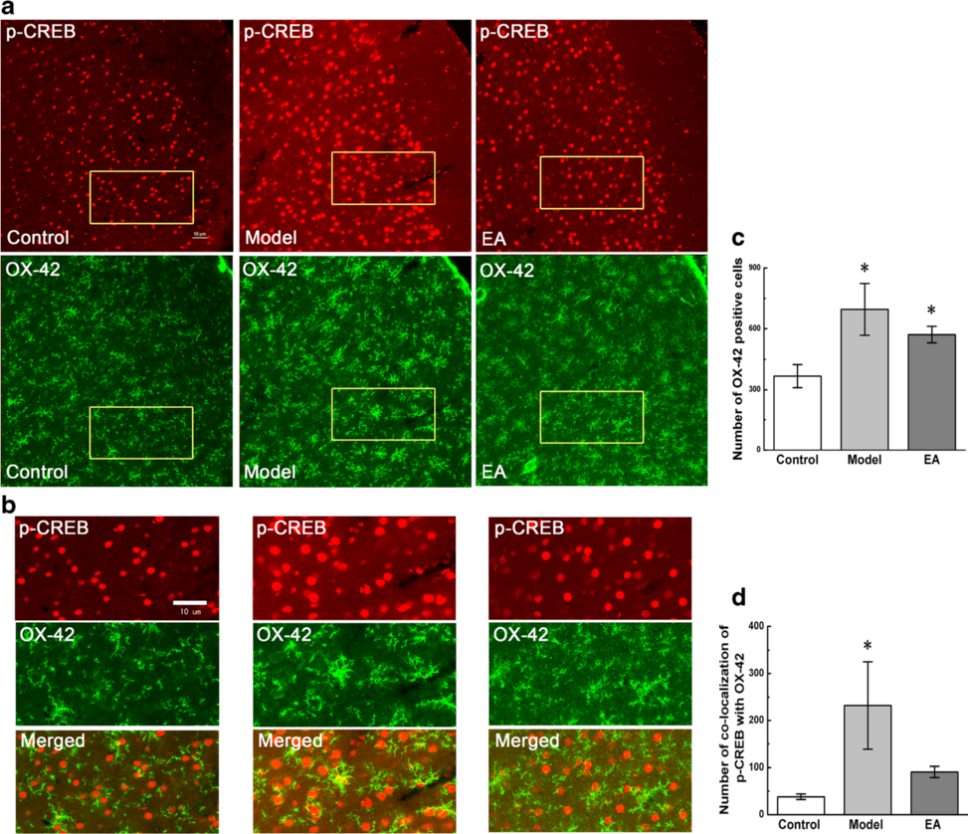
**Co-localization of p-CREB and OX-42 in coronal brain sections of ACC.** Photomicrographs showed the expression of p-CREB (red) and OX-42 (a microglial marker, green) from the same sections in figure **a**. Figure **b** was a high magnification image of the areas indicated by the yellow squares in the figure **a**. The double-immunofluorescence labeling showed that p-CREB co-expressed with OX-42 in the ACC. Numbers of OX-42-positive cells and co-localization of p-CREB with OX-42 were analysed in figure **c** and **d**. Error bars indicated standard error of the mean. Four rats for each group, five slides for each rat. * p < 0.05 vs. the control group.

Similar to GFAP, the increase in the number of OX-42-positive cells in ACC was observed both in the model group and EA group, compared with that in the control group (P < 0.05, Figure 5a, c). The double immunofluorescence showed the increased numbers of OX-42-positive cells co-expressing p-CREB in the model group (P < 0.05), and there was no obvious increase in co-localizations of p-CREB and OX-42 in EA group (Figure 5b, d), compared with those in the control group.

**Figure 6:**
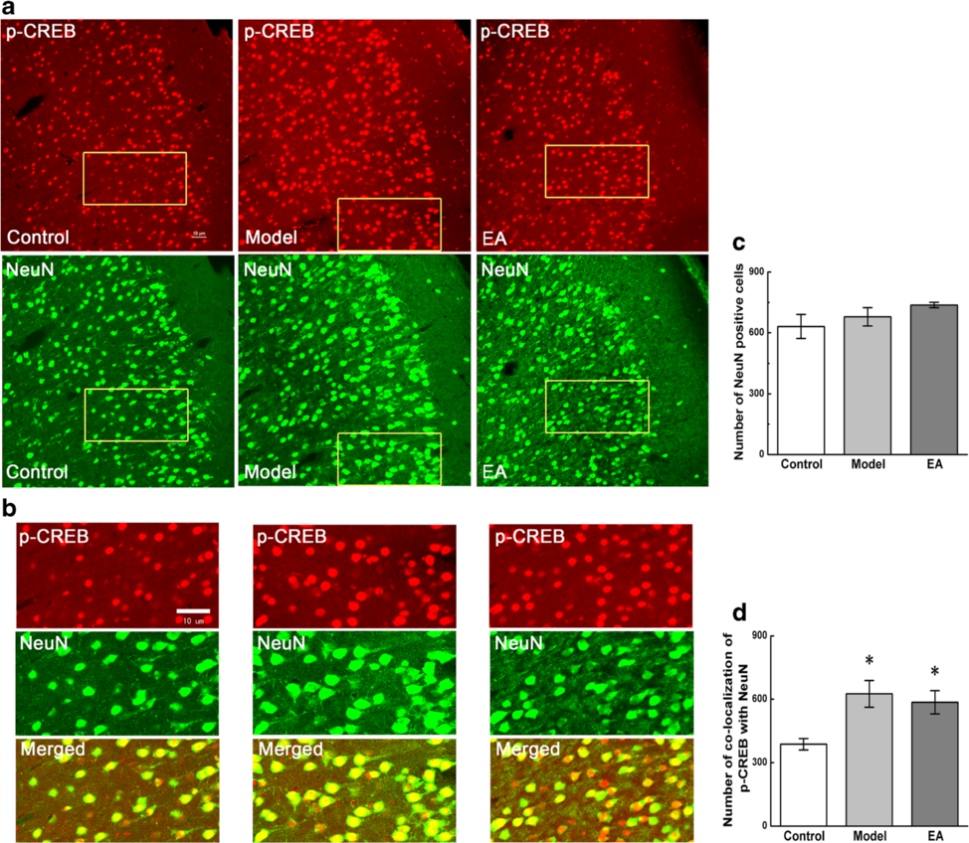
**Co-localization of p-CREB and NeuN in coronal brain sections of ACC.** Photomicrographs showed the expression of p-CREB (red) and NeuN (a neuronal marker, green) from the same sections in figure **a**. Figure **b** was a high magnification image of the areas indicated by the yellow squares in the figure **a**. The double-immunofluorescence labeling showed that p-CREB co-expressed with NeuN in the ACC. Numbers of NeuN-positive cells and co-localization of p-CREB with NeuN were analysed in figure **c** and **d**. Error bars indicated standard error of the mean. Four rats for each group, five slides for each rat. * p < 0.05 vs. the control group.

Compared with the control group, the number of NeuN-positive cells did not change in the model and EA groups, while the number of NeuN and p-CREB double-labeled cells increased significantly in both groups (P < 0.05, Figure 6a, c). EA group did not show any difference in NeuN and p-CREB co-localizations with the model group (Figure 6b, d).

## Discussion

In this study, we presented inspiring outcomes: (1) Pain memory occurred in model rats after the second carrageenan injection; (2) The EA treatment not only alleviated the initial pain induced by carrageenan injection, but also delayed the awaken time of pain memory and reduced the intensity of pain; (3) EA down-regulated the expression and ability of combination of p-CREB in ACC, inhibiting the retrieval of pain memory; (4) EA contributed to the decrease of the expression of p-CREB in the astrocytes and microglia cells, but not in the neurons, impacting the pain memory.

### EA alleviated pain by intervening in the retrieval of pain memory

Memory is a key variable for pain management [29]. The process of pain memory consists of acquisition, consolidation and retrieval [14,30-32]. Nociceptive stimulation acquired and transferred to related cortex becomes a long-term memory that is manifested in the increase of protein synthesis, changes of synaptic structure and function [28,33]. More importantly, expectation of pain established and consolidated through associative learning can lead to affective responses similar to those evoked by acute pain, as well as coping responses that modulate subsequent pain perception [34]. The Dr. Kissin’s experiment demonstrated that after recovery of hyperalgesia induced by the initial carrageenan-induced inflammation, repeated inflammation led to the development of a distant hyperalgesia that was absent during the initial inflammation [3]. In this study, we found that the second carrageenan injection did not only induce the similar acute inflammation pain in the injected hind paw, but also formed hyperalgesia in the previously-injected but non-post-injected hind paw from 24 h to 72 h after the second injection into the opposite hind paw. It evidences that the pain memory occurred successfully by carrageenan-injection twice. The acute inflammation pain following a single injection of carrageenan in rat’s hind paw of one side would be the process of acquisition the pain information, and transforms gradually into long-term memory which is consolidated by other environmental stimulations and factors.

At the same time, the EA treatment postponed the awaken time of pain memory to 72 h after injection and reduced the intensity of pain as compared to model group. However, the pre-administration of EA had no influence on the acute inflammatory pain caused by carrageenan-injection twice in the left hind paw. This suspects the possibility that different interventional mechanism is existed between pain memory and pain perception by EA.

### ACC took part in the pain memory by regulating the level of p-CREB

Several lines of evidence have implicated the critical role of ACC in the formation of pain memory. Electrophysiological recordings from ACC neurons in animals showed that ACC cells responded to peripheral noxious stimuli [28,35]. Neuroimaging studies in humans had further confirmed that the ACC, together with other cortical structures, were activated by acute noxious stimuli, psychological pain, and social pain [36,37]. The ACC was also considered as a pivotal region for emotion that involves events, memories, and association with autonomic changes. Perigenul ACC activation was involved in long-term negative affective state and prediction of aversive stimuli by contextual cue for the memory processing [8]. There exists strong evidence that long-term changes in the ACC contributed to chronic pain-induced cognitive and emotional impairment assessed by using behavioral test and electrophysiological approaches in mice [13,38]. Therefore, ACC is a nucleus in brain that is closely related to pain and pain memory.

The pain itself was closely related to CREB activation and the interrelation between them can be effectively intervened by EA therapy [39]. More importantly, CREB plays a central role in the formation of long-term memory [40-42]. Various reports demonstrate that the connection of CREB and long-term memory is definite. The researches reported that the induction of a dominant-negative mutation or the deletion of key CREB isoforms blocked long-term memory [11,43]. And the increased level of p-CREB at Ser-133 resulted in the synaptic plasticity in the process of learning and memory [44,45]. Furthermore, there is considerable evidence indicated that the expression of certain genes through phosphorylation of CREB is required to maintain the late phase of long-term potentiation and long-term memory [46,47]. Together with all mentioned above, recent studies have shown that both ACC and p-CREB played an important role in pain and its memory. That is why we have chosen both of them for exploring the effect and its mechanism of EA treatment in intervening pain memory.

### EA down-regulated the expression and activity of combination of p-CREB in ACC to inhibit the retrieval of pain memory

The results of this research indicated that phosphorylation of CREB increased significantly, and could be effectively inhibited by EA treatment. The other studies demonstrated that disruption of CREB function impaired both memory for objects and memory for spatial location of objects after a 24 h delay [12,48]. In the adult rat, CREB is normally abundant in its non-phosphorylated state. When its upstream signaling kinases are activated in response to environmental stimulations and factors such as stress, mitogens, and excitatory signals including calcium, CREB becomes phosphorylated [49,50]. Meanwhile, the increased expression of p-CREB is a manifestation of the formation of memory in the cerebral cortex in rodents [51]. Therefore, it is a memory of pain that aggravates the response to pain caused by phosphorylation of CREB, and the EA treatment in the study may act as an analgesic by decreasing p-CREB.

When CREB is phosphorylated at Ser 133, it promotes the transcription of genes by interacting with the CRE sequence located in the promotor regions (TGACGTCA) [52-54]. The EMSA indicated the ability of this combination. In experiment’s results, the amount of DNA-protein complex was gradually increased in the rats of the pain memory model. However, p-CREB binding was decreased in the rats of EA group. This revealed that EA could lower the level of activated combination between p-CREB protein and its DNA. Other research suggested that the key steps involved in CREB-mediated gene transcription included dimerization, binding at response elements in DNA, and phosphorylation [43]. By using immunoelectronmicroscopy, immunoblotting, and electrophoretic mobility shift assays, Cammarota et al. strongly suggested that the activation of nuclear CREB might be related to the up-regulation of transcription during memory formation [55,56]. Some other reports also determined that p-CREB isolated from cultured cells could bind to an oligonucleotide identical in sequence to the CART gene promoter CRE cis-regulatory element in EMSA/super shift analyses [57,58]. These results suggested distinctly that memory of pain was generated in response to the improved ability of an activated combination between p-CREB protein and its DNA. On the contrary, EA treatment could inhibit it to partially block the memory of pain. This indicates that EA treatment is able to intervene in the combining capacity of p-CREB protein and CRE-containing oligonucleotide. Above results of p-CREB in ACC suggests that EA treatment has a positive mechanism of its effect on pain memory.

### EA intervened in the expression of p-CREB in the astrocytes and microglia cells in ACC to impact the pain memory

To distinguish the target nerve cells in the ACC that participated in the effect of EA on pain memory in rats, we observed the expression of p-CREB in neurons (NeuN) and gliocyte which including astrocytes (GFAP) and microglia (OX-42) by double-immunofluorescence labeling. The results showed that EA intervened in the expression of p-CREB in the astrocytes and microglia, but not in the neurons in ACC of the pain memory model.

Relevant studies demonstrated that activation of astrocytes in the ACC contributed to the affective component of pain [59]. Other research confirmed that in the condition of inflammatory pain, astrocytes were converted to an activated state and amplified the magnitude of LTP [13], and that LTP in the ACC was a mechanism for persistent affective changes in patients with chronic pain [60]. Our results showed the consistency with previous researches. We found in the model of pain memory, astrocytes could be activated because of the retrieval of pain memory. At the same time, the increase of the expression of p-CREB in the astrocytes of model group was significant and EA could inhibit this trend. EA treatment significantly declined the expression of p-CREB in astrocytes. This indicates EA might inhibit pain-induced central sensitization, via intervening in the activation of p-CREB in astrocytes, which is resulted in a reduction in the development of pain memory.

Few studies have investigated whether microglia are involved in pain sensitization above the spinal cord. However, in the development and maintenance of the chronic pain state, microglia may be activated without any changes in obvious morphological or staining characteristics, which have previously been used to characterize the resting-to-activated transition. It appears that even in the resting state, microglia is functionally active [61]. The results of this study demonstrate the numbers of p-CREB expressed in microglia were increased obviously in the ACC and EA therapy had a tendency to inhibit.

It is well accepted that neuron and glia always interact together in brain functions and neurological disorders including chronic pain [62-64]. By using electrophysiological approaches, the study provided strong evidence for long-term changes of temporal precision of information coding in cortical neurons after peripheral injuries and explained neuronal mechanism for chronic pain caused cognitive and emotional impairment [38]. In this study, though the number of neurons in three groups didn’t show the difference, the expression of p-CREB in neuron increased in the model group. It demonstrated that p-CREB expressed in neuron had participated in the process of pain memory. EMSA result also suggested the decline of activity of p-CREB owned to the EA intervention. So it could be inferred that EA might intervene in the pain memory in neuron by declining the activity of combination of p-CREB, without affecting the expression of p-CREB in neurons. Moreover, that whether there are other factors or signaling pathways exchanges in neurons of pain memory, needs to be explored in the future. Besides, astrocytes outnumbered neurons and were closely associated with neurons [65]. More researches must be done to find whether or not neurons interact with astrocytes or microglia and the mechanism of EA intervention is possibly related to the change in neurons.

## Conclusions

In conclusion, the present study demonstrates that inflammation-induced pain memory could be indeed caused by repeated injections of inflammatory mediators in rats. EA stimulation is able to influence pain memory by delaying its awaken time and reducing the intensity of pain. The pain memory and its retrieval are most likely related to the increase of p-CREB in ACC. The possible mechanism of EA on alleviating pain memory is at least partially related to the inhibition of the expression of p-CREB in astrocytes and microglia in ACC. Therefore, EA treatment might be a potential pathway of intervention in the induction and development of pain memory.

## Abbreviations

cAMP: cyclic adenosine monophosphate
p-CREB: phosphorylation of cAMP response element-binding protein
ACC: Anterior cingulate cortex
EA: Electroacupuncture
PWTs: Paw withdrawal thresholds
IF: Immunofluorescence
EMSA: Electrophoretic mobility shift assay
ANOVA: Analysis of variance
LTP: Long-term potentiation
LSD: Least significant difference
